# On the Treatment of Fever

**Published:** 1848-03

**Authors:** 


*On the Treatment of Fever.* By A. G. Henry, M. D., of Pekin, Illinois.

Dr. Henry communicated, some time ago, a novel method of treating fever with large doses of opium, by which he affirmed the disease may generally be arrested *in limine.* We find, in the *Illinois Journal,* a paper by him on the pathology and treatment of fever, which was read before the Peoria District Medical Society, and ordered to be published in that journal. We omit his remarks on pathology of fever, and present only the portion of the article which relates to the treatment:—

"Having established (at least to my own satisfaction) that fever is the result of disease in the nervous system, and that all other derangements are to be regarded as secondary affections, I shall proceed briefly to give my views of the practice which ought to be pursued in the commencement of the attack, and I know of no way by which I can make myself understood more clearly, than by reporting one or more cases (out of the hundreds I have treated on this plan, with the same uniform success,) of the different forms of fever found in this region of the country, during the last ten years.

"William S——, laborer, was attacked with fever in February last. I saw him a few hours after he took to his bed at about nine o'clock in the evening. He had been complaining of languor and debility during the day, but did not leave his work until four in the evening, when he was suddenly seized with violent rigors, and so great was the muscular prostration, that he was unable to stand without help. He complained of general soreness over the whole surface. Pulse over 140, full, but easily compressed, with quick breathing, interrupted occasionally with a long, heavy sigh ; skin hot, but not dry. Complained of no pain except a slight pain in the head. Could fetch a full inspiration without causing pain. I opened a vein in the arm, more for the purpose of seeing the effect it would have upon the breathing, than from any expectation that it would relieve the symptoms ; and after taking about 16 ounces, the symptoms were evidently aggravated. As usual, in such cases, I gave him five grains of quinine, with five pills composed of one grain of opium and three grains of calomel, each, and left a powder composed of five grains of quinine with three of opium, to be given in six hours. I saw him two hours after, and found him sweating freely ; pulse about 100, strong and full ; breathing natural, and said he felt entirely well. Saw him the next morning at eight ; had taken the powder as directed, and sweat freely during the night ; pulse natural, pain and soreness gone ; had not slept much during the night. I now gave him salts and senna, which operated freely in three or four hours, when I repeated the opium and quinine in doses of three grains of each every six hours, until three doses had been taken. In the evening, found him sitting by the fire comparatively well, but complained of feeling extremely weak. Directed him to keep in doors for two days and live on soups, tea and toast, and at the end of that time he was at his work again as though nothing had happened. Some ten other cases of this kind were treated in this way, except the bleeding, with the same results in the course of eight or ten days, during which time, five cases proved fatal under the evacuant plan of treatment in other hands in forty-eight hours, becoming entirely insensible after twenty or twenty-four hours from the commencement of the attack. The only patient I lost, was a child five years old. In this case, I gave a dose of ipecac to relieve the difficult and rapid breathing. I returned in two or three hours after, and found my patient beyond the reach of remedy, and she died in nineteen hours from the first symptom of indisposition, which was a slight chill, followed with fever and rapid breathing.

"I regarded these cases as most violent and malignant forms of Typhus fever, or, as they are sometimes called, cases of Typhoid Pneumonia, Cold Plague, Winter Fever, &c., differing only from the milder forms of Typhus, in the fact of being more rapid in their progress in consequence of the nervous system being, as it were, *overwhelmed by the force of the exciting cause.* Hence the necessity or rather the advantage of combining the quinine with the opium and calomel. The quinine might have been omitted with safety, but in such cases I prefer using it. The calomel I know can be dispensed with without lessening the efficacy of the treatment, as I have demonstrated over and over again in the treatment of this form of fever, and I only give it as a matter of convenience, for its cathartic effect. The following case is in point. I would remark here, that I never resort to the quinine except in the malignant forms of the disease.

"I was called to see Mrs. H——, who had recently lost in her family, a nephew and a daughter, after a lingering illness of what the doctor called the winter fever, both of which had been treated on the mercurial plan. She had been confined to her bed for five days, but had taken nothing but oil and pills of some kind. There was every symptom of Typhus Fever present, with slight affection of the lungs. She begged that I would not give her calomel, and not regarding it material, I promised I would not. I accordingly left her two powders of four grains of opium each, the second to be given eight hours after the first, and to drink hot sage tea freely until sweating was produced. Saw her the next day, much improved in appearance, and said she felt much better—had perspired freely all night. Directed a dose of oil and four grains of opium at bed time, with *three* grains eight hours after, with the same effect as before. Found her the next day greatly improved, and entirely free from fever. Repeated the oil, and left three grains of opium to be given at bed time. Did not see her again for three days. Found her sitting up free from fever, but very much troubled with a dry cough. Applied a blister to the chest, and left her the brown mixture. She recovered steadily but slowly, the cough not leaving her for ten or twelve days. Several other cases occurred in the same neighborhood, which I treated with opium in the same way, with the addition of the calomel, all of which were convalescent in forty-eight hours after the first visit, and no case was seen after the third visit.

"Five cases, treated by other physicians, proved fatal in the neighborhood that fall and winter, (last winter). I am certain that I prescribed for two-thirds of all the cases that occurred, without losing a single case of fever in the vicinity.

"I have now given a case of the mild, and most malignant forms of *Western* Typhus Fever. I now propose to give, for the purpose of more clearly illustrating my system of practice, one case of Congestive Intermittent, and one of common Remittent.

"Some three weeks since I was called to see Mrs. S.——. She had been seized the evening before with what she supposed to be a simple chill and fever. The chill had lasted some six or eight hours, accompanied, as she expressed it, 'with a burning fever, and a feeling of freezing all the time,' with the sensation of a loaded waggon running over her breast. She was lying in bed comparatively comfortable, and free from fever as she supposed, but expressed herself as alarmed for the consequence of the next chill, which she was expecting in the course of two hours. On feeling her pulse I readily detected that peculiar, but indescribable pulse, always found during the intermission in this form of disease. She said she felt tired, and when she attempted to sit up, she became sick at the stomach. I was satisfied from her general appearance that the next paroxysm would prove fatal, unless prevented or greatly mitigated, and there was no time to be lost. Accordingly, I administered in a little coffee, five grains of quinine with five of opium, and left five more of quinine with two of opium, to be given in six hours, directing her to drink hot tea freely until she got into a free perspiration, but before leaving the house she complained of feeling cold, and commenced breathing quick and laboriously. On going to the bed side, I found her hands cold; pulse small and rapid. The powder had only been swallowed some fifteen minutes, not long enough to produce any sensible effect upon the system, still I had every confidence that it would act in time to moderate the paroxysm. I applied a strong mustard poultice to the stomach, which acted promptly, when the breathing improved, but by this time the powder began to act. The pulse became slower and more distinct, drops of perspiration showed themselves upon the forehead, and in one hour from the time the dose was administered, she was perspiring freely, and said she felt better than she had for two days before; and being satisfied that she was safe, I retired for the night, repeating the direction to have the second powder given at the end of six hours. The next morning I found her entirely comfortable—had not slept much during the night, but felt disposed to sleep then. I directed a dose of oil, which operated in two hours very freely, when I repeated the quinine in five grain doses without the opium, and left my patient convalescent. The next day she was up doing her work.

"I am convinced that if the dose had been delayed one hour longer in this case, the paroxysm would have proved fatal, for I am satisfied from experience, that but little can be effected by way of mitigating the paroxysm after it is once fully formed, by internal remedies. If there is vigor enough in the vis medicatrix naturæ to throw it off, you can save your patient by rightly improving the remission. I say *remission*, because I am satisfied that we have no *intermission*, such as we have in our ordinary intermittents, in this malignant form of fever, and I am equally satisfied that nothing but sedative doses of opium or quinine will save our patients—and of the two I prefer opium, for five grain doses of it prevents the paroxysm certainly, while the largest doses of quinine sometimes fail, particularly where the stomach is inclined to be irritable, which is often the case ; and for the further reason, that the opium exerts no deleterious influence upon the system, which cannot be said with truth of ten or fifteen grain doses of quinine. From five to six grain doses of quinine, will, for all time to come, be my maximum dose. If I desire a sedative effect, I can procure it with more certainty with opium in four or six grain doses*; which I contend is the smallest dose that can be relied upon for the full sedative effect required for a case of fever.* The reason why so little benefit has been obtained from opium in the treatment of fever, heretofore, is to be attributed to the fact of its never having been given in full doses until given by myself.

"When Dr. Cartwright, of Natchez, Miss., first proposed ten grain doses of quinine, the Medical World were as much shocked with its absurdity, as they now possibly can be at the dose of opium which I recommend ; and it is but recently that it is conceded that it can be given in ten grain doses with comparative impunity, and to the extent of forty and fifty grains in malignant forms of disease, without being destructive to life—and it is now universally admitted, that we can obtain two opposite effects by regulating the quantity. If we give it in one grain doses, repeated every hour, in a paroxysm of fever, we aggravate all the symptoms, and in all probability, before ten grains shall have been given, fatal congestion of the brain will have been produced. But if were to give the ten grains at a dose, in precisely a similar case, none of these consequences will follow, but on the contrary we shall obtain full relief to all the symptoms by subduing the fever. This principle is more particularly applicable to opium than to quinine. Medical writers have long suspected that opium had an action different from that which had been usually attributed to it, and among them I would name the justly celebrated Pereira. [See his Materia Medica under the head of opium.] But no one, before myself, has had the temerity, as it has been called, to demonstrate the truth of the proposition by actual experiment upon the human system, in the treatment of a form of disease that occupies four-fifths of the time and talent of the Medical World ; but when the practice shall take the place of the present abused mercurial system, (as it is bound, sooner or later, to do,) there will be a saving of thousands of lives, and an incalculable amount of human suffering.

"But the great obstacle in the way of its adoption by the Profession, as I have said before, is the dread of fatal congestion of the brain, if given in such large doses. One says he has known fatal congestion of the brain, to follow the use of *one grain*, repeated once in two or three hours, until four or five grains had been taken ; and they exclaim with uplifted hands and eyes : 'My God ! if such results follow from one grain doses, what, but almost instant death, could be expected from a five grain dose.' My friend, Dr. Golliday, in his learned 'critique' upon a communication of mine, published in the Illinois and Indiana Medical Journal, some year since, on the treatment of fevers and inflammations without mercury, in which I urged the use of from four to six grains of opium, quotes various authorities to prove that my practice is not admissable in inflammatory affections, although it cannot be anywhere shown 'in the writings of Dr. Christison, Dr. Sobernheim, Dr. Rankin, Dr. Williams, or Dr. Pereira,' that they had ever used a four or five grain dose of opium in their lives, in the treatment of diseases alluded to, in which they condemn its use, when I claim to have given it hundreds of times in these doses with the happiest effects.

"Now I would ask the Doctor how he can give the preference to their authority based on mere theory, without calling in question my veracity, a thing which I presume he had no intention of doing.

"The Doctor does not seem to be aware of the fact, that I contend as strenuously as any other man, that anything short of a *full sedative dose*, (and by this I mean from four to six grains,) will as a general thing, do positive injury ; and in that very communication which he very properly took the liberty of criticising, I laid down the proposition, that a two grain dose repeated once in two or three hours, was almost sure to do mischief, when a five grain dose given once or twice in twenty-four hours, would be productive of great benefit. As in the case with quinine, so with opium. Give it in one grain doses in a paroxysm of fever, every hour until four or five grains have been given, and the chances are two to one that you will greatly injure, if you do not destroy your patient. But give it in one dose, and none of these unpleasant consequences follow.

"I have already extended my remarks far beyond anything I had intended in the outset. One word in relation to remittents, and I am done.

"John R———, a German laborer, recently arrived in the country, was taken down the first of September with all the symptoms of a common bilious remittent. When I saw him he had been in bed 24 hours with high fever, hot, dry skin, great thirst, and violent pain in the head and back. I found him the second day about noon, with all these symptoms aggravated ; pulse 120, full and tongue dry and glossy, and very red at the point. Bowels had not been moved for thirty-six hours. I gave him an emetic of Ipecac in broken doses, at long intervals, say half an hour, for the purpose of securing a full emetico-cathartic effect. Saw him four hours after and found that his stomach and bowels had been thoroughly evacuated, but with little relief to the symptoms. I now gave him five pills composed of opii i grain, calomel iii grains each ; directed him to drink freely of hot tea, and left him for the night with every confidence of finding him in the morning entirely relieved. I found him as anticipated, free from fever, the pain in the head and back entirely gone, tongue moist and heavily coated with a loose, white fur. Said he got easy about two hours after taking the pills, and had sweat freely all night, and expressed himself as feeling almost well. Left him three five grain doses of quinine, to be taken once in four hours during the day, and to drink as much water gruel as he pleased. Found him in the evening down at the table eating his supper; said he was very hungry, and felt entirely well, 'except a little weak.' His bowels had been freely moved during the day by the calomel. On the 4th of September he was again at his work as usual.

"I have selected this case from among many others treated in the same way, for the reason that he complained more of his head and back than any other case I have treated this season, by way of showing Dr. Golliday and others, that a five grain dose of opium can be given, not only with safety, but with decided advantage, even when there was 'strong symptoms of congestion of blood,' and that too, to the brain and spinal column.

"Now, supposing this case had been treated upon the usual plan; bleed first, give the emetic and the calomel at night without opium, does any gentleman suppose that he would have passed a comfortable night, been entirely free from fever in the morning, and at his work the next day? Would it not have been more likely that he would have spent a distressing night, with a temporary intermission in the morning, to be followed with an exacerbation in the evening, and this repeated day after day until mercurial action was established, or he beyong the reach of all remedy. Such has been the result of similar cases treated on the mercurial plan, at the *same time, and in the same neighborhood*, and such would have been the result had he fallen under my treatment ten years ago.

"Do not such results go to prove beyond all question, that large doses of opium possess a power in the treatment of fever, greater than any other known remedy. The remedy which I once regarded as my sheet anchor, I can now dispense with entirely without material detriment to the treatment. But I do not propose to throw it aside; on the contrary, I still regard it as one of the best and most cenveniant *purgatives* in the whole materia medica I only ask that it shall no longer be relied upon as the main remedy in the treatment of fever; for the reason that we have, in large doses of opium, one better adapted to meet the indication of cure.

"In conclusion let me caution those of my professional brethren who would give the remedy a trial, not to delay a resort to it *until they have exhausted every other known remedy*, and when the case is beyond the reach of human agency. If you use it at all, use it in the early stage as I have directed, and my word for it you will not find cause to regret it."

